# Function and mode of action of cytohesins in the epidermal growth factor pathway in colorectal cancer cells

**DOI:** 10.3892/ol.2012.1064

**Published:** 2012-12-05

**Authors:** TAO PAN, JUNFENG SUN, JUN ZHOU, ZHIXUAN FU, YIWANG HU, SHU ZHENG, SUZHAN ZHANG

**Affiliations:** Cancer Institute, The Second Affiliated Hospital, Zhejiang University School of Medicine, Hangzhou, Zhejiang 310009, P.R. China

**Keywords:** epidermal growth factor receptor, cytohesin, colorectal cancer, pathway, ARNO

## Abstract

Cytohesins have been identified as cytoplasmic ErbB receptor activators in certain cancers, exhibiting an important role in ErbB signaling. However, whether cytohesins are essential in colorectal cancer is unknown. The aim of the present study was to investigate whether cytohesins contribute to the epidermal growth factor (EGF) pathway in colorectal cancer cells. RT-PCR and immunofluorescence experiments were employed to detect the expression of cytohesins in colorectal cancer cell lines. The EGF pathway activation conditions were investigated by examining the phosphorylation of the epidermal growth factor receptor (EGFR) and intracellular signal-related kinases, with or without chemical inhibition (SecinH3) and knockdown of cytohesins. An MTT assay was conducted to examine the inhibitory effect of SecinH3 and cytohesin-specific siRNA in HT-29 cells. Results demonstrated that the four homologous members of the cytohesin family were expressed in the four colorectal cancer cell lines. Notably, a significantly higher expression level of cytohesin-2 (ARNO) compared with the other three homologous family members was observed. Stimulation with EGF and SecinH3, as well as knockdown of ARNO, are capable of reducing EGF pathway activation and proliferation of HT-29 cells. In conclusion, cytohesins play an essential role in the activation of the EGF pathway and may be a potential target in colorectal cancer therapy.

## Introduction

Colorectal cancer is one of the most common clinical gastrointestinal cancers that poses a serious threat to human health. The GLOBOCAN 2008 estimates stated that colorectal cancer is the third most commonly diagnosed cancer in males and the second in females, with over 1.2 million new cancer cases and 608,700 fatalities occurring every year (1). Effective treatments for colorectal cancer include surgery, chemotherapy and targeted therapy. Chemotherapy and targeted therapy are the final strategies implemented to extend patient survival, particularly with advanced metastatic colorectal cancer (2,3). However, present evidence-based medicine indicates that colorectal cancer patients hardly benefit from chemotherapy due to chemical toxicity and self-resistance. Therefore, targeted therapy has a greater potential and is currently being investigated further, with a greater research emphasis on cancer therapeutics. Throughout the past decade, targeted treatment of cancer has mainly focused on the epidermal growth factor (EGF) pathway (4,5).

The epidermal growth factor receptor (EGFR) is a key member of the ErbB family, which consists of four members: EGFR (ErbB1), Her2 (ErbB2), Her3 (ErbB3) and ErbB4. In cancer cells, the extracellular domain of the EGFR binds to the EGF and the EGF pathway is activated; signaling is initiated to regulate the differentiation, survival, proliferation and migration of cancer cells (6). However, activation of the EGFR is required for signaling initiation; the ligand-induced conformational change in the receptor ectodomains results in the association of the cytoplasmic tyrosine kinase domains of two receptor molecules (7). The activation of the pathway depends not only on EGF as the ligand binding to the EGFR ectodomains, but also on the activation of homodimerized or heterodimerized cytoplasmic domains of EGFRs (8,9). Bill *et al* have identified cytohesins as conformational activators of the cytoplasmic dimer, which play an important role in lung cancer ErbB signaling (10).

The cytohesin family includes four highly homologous members: Cytohesin-1, -2 (ARNO), -3 (Grp1) and -4 (11). Cytohesins are guanine nucleotide exchange factors (GEFs) for ADP ribosylation factors (ARFs) that belong to the family of small Ras-like GTPases. As with the case of other small GTPases, ARF function critically depends on activation by GEFs (12). Therefore, cytohesins are important regulators of cytoskeletal dynamics, cell migration, vesicular traffic and signaling (10,11,13).

Bill *et al* demonstrated that cytohesin overexpression increases EGFR activation and signaling. Moreover, siRNA and chemical inhibition of cytohesins produced consistent results both *in vivo* and *in vitro* in human lung adenocarcinomas. Therefore, the authors concluded that cytohesins were conformational activators of the ErbB receptor in lung cancer (10). In the present study, we demonstrated that EGFR signaling was reduced when cytohesins were inhibited in the HT-29 cell line. Subsequently, whether cytohesins have the potential to act as a target for colorectal cancer therapy was preliminarily investigated.

## Materials and methods

### Reagents

Cell culture media included RPMI-1640, McCoy’s 5A and L-15, which were purchased from Genom (Shanghai, China). The following mouse anti-human antibodies were used: Cytohesin-2 (cat. no. ab56510; Abcam, Hong Kong, China); p-EGFR (pY1068, cat. no. 1138-1; Epitomics, Burlingame, CA, USA); p-ERK1/2 (T202/Y204, cat. no. BS5016; Bioworld Technology, Inc., St. Louis Park, MN, USA); EGFR (cat. no. 3197; Cell Signaling Technology, Inc., Danvers, MA, USA); GAPDH (cat. no. AP0063; Bioworld Technology, Inc.); phycoerythrin (PE)-conjugated rabbit anti-mouse IgG and fluorescein isothiocyanate (FITC)-conjugated goat anti- rabbit IgG (cat. no. GAM007; Multisciences, China). TRIzol RNA Isolation and M-MLV RTase kits were purchased from Promega Corporation (Madison, WI, USA), and the Real-Time PCR kit was purchased from Fermentas (USA). SecinH3 (cat. no. 565725/sc-203260) was purchased from Merck and siRNA oligo was purchased from Shanghai Gene Pharma (China). The following reagents, 3-(4,5-dimethylthiazol-2-yl)-2,5-diphenyl tetrazolium bromide (MTT; cat. no. m5655), dimethyl sulfoxide (DMSO; cat. no. D5879) and 0.25% trypsin, were purchased from Sigma (St. Louis, MO, USA). Human EGF (cat. no. AF-100-15) was purchased from Peprotech, Inc. (Rocky Hill, NJ, USA) and fetal bovine serum (FBS) was purchased from Gibco (Carlsbad, CA, USA).

### Cell lines and cultivation

Human colorectal cancer cell lines including HT-29, SW620, SW480, LOVO and HCT-116, were obtained from the Key Laboratory of Cancer Prevention and Intervention, Cancer Institute, Second Affiliated Hospital, School of Medicine, Zhejiang University, China. The HT-29 cell line was cultured in RPMI-1640 (with 10% FBS and 1% streptomycin/penicillin); SW620, SW480 and LOVO cell lines were cultured in L-15 (with 10% FBS and 1% streptomycin/penicillin); HCT-116 cell line was cultured in McCoy’s 5A (with 10% FBS and 1% streptomycin/penicillin). All cell lines were cultured at 37°C and 5% CO_2_ in an incubator, and passaged with 0.25% trypsin (Sigma) in 0.2 mol/l phosphate-buffered saline (PBS; Sigma). The study was approved by the ethics committee of the Cancer Institute, The Second Affliated Hospital, Zhejiang University School of Medicine, Hangzhou, China.

### RT-PCR

Primers were designed according to the Genbank sequences and were synthesized by Shanghai Sangon (Shanghai, China). The primer sequences were as follows: Cytohesin-1, 5′-AGTGCATTAAAGCAGCCATCAG-3′ and 5′-TCAGTGTCGCTTCGTGGAG-3′; cytohesin-2 (ARNO), 5′-GAAACCGAACTGCTTTGAACT-3′ and 5′-CAGCCGCCTGATGGACT-3′; cytohesin-3 (Grp1), 5′-ATG AAATCCATCAAAGCCAGTA-3′ and 5′-CAATCCTT CGTTTCCTCGTT-3′; cytohesin-4, 5′-GTCCATCCGAGCC AGCAT-3′ and 5′-GGTAACGGGGAACAGCAAT-3′; GAPDH (human housekeeper gene), 5′-AATGTGTCCGTCGT GGATCTG-3′ and 5′-CAACCTGGTCCTCAGTGTAGC-3′. Total RNA was extracted using the TRIzol RNA isolation kit and cDNA was synthesized using the M-MLV RTase kit, according to the manufacturer’s instructions. For this reaction, GAPDH acted as an inner control and was amplified in each reaction system. The reaction conditions were 95°C for 3 min, 40 cycles of 95°C for 10 sec, 62°C for 35 sec and 72°C for 60 sec.

### Immunofluorescence

Aseptic slides were placed in 24-well plates and after prewarming at 37°C for 24 h, 10^4^ cells/well from the HT-29 cell line were incubated in the plates. Cells were cultured with RPMI-1640 culture medium at 37°C and 5% CO_2_ in an incubator until cell growth covered 60–80% of the slides. Then, the culture medium was removed and cells were fixed in 4% paraformaldehyde for 15 min. After washing three times with PBS and 0.25% Triton X-100/TBS for 10–15 min at room temperature, mouse anti-cytohesins IgG were incubated overnight at 4°C. Following repeated washing with PBS, slides were incubated with PE-conjugated rabbit anti-mouse IgG and FITC-conjugated goat anti-rabbit IgG as secondary antibodies for 1 h at 37°C, then washed with PBS and coverslipped. Subsequently, ARNO and EGFR expression was observed using a Zeiss LSM-710 fluorescent microscope with a Spot digital camera (Carl Zeiss, Germany). For comparable analysis of the intensity levels of ARNO and EGFR expression, the same exposure conditions were maintained throughout the experiment.

### Western blot analysis

Cells were collected and extracted by the eukaryotic cell lysis buffer according to the manufacturer’s instructions (Total protein extraction kit 2140, Merck Millipore, Billerica, MA, USA). Then, proteins were separated by 12% SDS-PAGE and blotted to a nitrocellulose membrane by a wet transfer device (Bio-Rad, Hercules, CA, USA). Blotted membranes were blocked by 10% skimmed milk in PBS Tween-20 (PBST) for 1 h. After washing three times with Tris-buffered saline Tween-20 (TBST), membranes were incubated with primary antibody diluted 1:1,000 at room temperature for 1 h, then incubated in HRP-labeled secondary antibody diluted 1:10,000 at room temperature for 1 h. After rinsing, visualization was conducted using the enhanced ehemiluminescence (ECL) western blotting detection system (Amersham Biosciences, Little Chalfont, UK) and cells were exposed to X-ray film (Kodak, USA). GAPDH protein was used as an inner control.

### siRNA selection

Three pairs of ARNO siRNAs were designed and synthesized by Genepharma Company (China). The siRNA sequence pairs were as follows: siRNA-1, 5′-GUUCU UGGUGGAGAAUGAATT-3′ and 5′-UUCAUUCUCCACC AAGAACTT-3′; siRNA-2, 5′-AGGCCCUCAGGCAGUUU CUTT-3′ and 5′-AGAAACUGCCUGAGGGCCUTT-3′; siRNA-3, 5′-GCUGGUUUAUCCUCACAGATT-3′ and 5′-UCUGUGAGGAUAAACCAGCTT-3′. Each pair of siRNA sequences was identified in the HT-29 cell line; cells were transfected with 100 pmol of each siRNA in 5 *μ*l Lipofectamine 2000/10^5^ cells/ml, and then cultured in serum- free medium. After 24 h, cells were collected for western blot analysis.

### MTT

HT-29 cells were plated in 96-well plates with a density of 3,000 cells/well. Cells were cultured with 1% FBS and inhibitors (20 *μ*mol/l SecinH3 or 50 nmol/l per 5 pmol ARNO/negative siRNA in 0.25 *μ*l Lipofectamine 2000) for 24, 48 and 72 h, at 37°C and 5% CO_2_. Then 5 mg/ml MTT (20 *μ*l) was added to each well and incubated for 4 h. Then, 200 *μ*l DMSO was added to resolve the MTT substrate and absorbance was measured at 570 nm using a SpectraMax Microplate Reader (Bio-Rad).

### Statistics

Results are presented as the mean ± standard error of the mean (SEM). The Statistical Package for the Social Sciences (SPSS) 16.0 software (SPSS, Inc., Chicago, IL, USA) was used for statistical analysis. Paired comparisons were performed using a Student’s t-test. P<0.05 was considered to indicate a statistically significant difference between means.

## Results

### All four cytohesins were transcribed and ARNO was expressed in colorectal cancer cells

**Figure 1.**
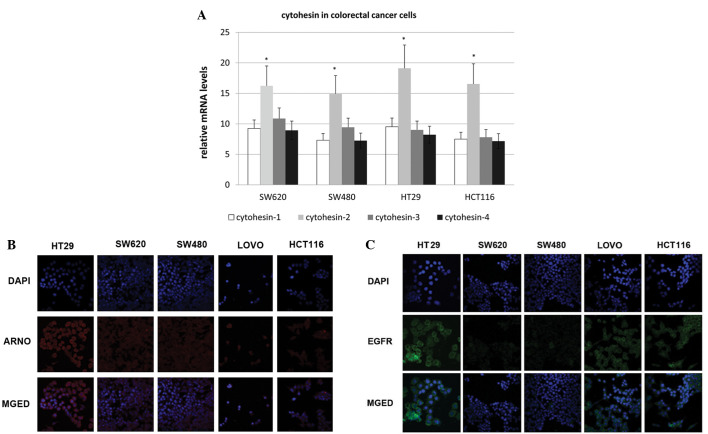
(A) The expression of cytohesin-1, -2 (ARNO), -3 and -4 mRNA in the four colorectal cancer cell lines. ^*^P<0.05 for cytohesin-2 vs. other cytohesins; n=3. (B) The expression of ARNO in colorectal cancer cells as detected by immunofluorescence experiments. ARNO expression is evident in the HT-29, SW620, SW480, LOVO and HCT-116 cell lines. The expression of ARNO is greatest in HT-29 cells (×200). (C) The expression of EGFR in colorectal cells as detected by immunofluorescence. EGFR expression is demonstrated in the HT-29, SW620, SW480, LOVO and HCT-116 cell lines. EGFR expression is greatest in HT-29 cells. Nuclei were stained with DAPI (×200).

RT-PCR was employed to detect the transcription of the cytohesin family. Cytohesin-1, -2 (ARNO), -3 (Grp1) and -4 were transcribed in all four cell lines, which included HT-29, SW620, SW480 and HCT-116. We found that mRNA of the four cytohesins was transcribed in all four cell lines, and ARNO mRNA had the highest expression level (Fig. 1A). Additionally, by an immunofluorescence assay, we demonstrated that ARNO was highly expressed in HT-29 cells and was located in the cytoplasm, near to the membrane (Fig. 1B). Therefore, the expression of EGFR in colorectal cells was detected by immunofluorescence (Fig. 1C). The expression of EGFR in HT-29 cells was higher than that of the other cell lines. Therefore, the HT-29 cell line was selected for EGF pathway research in the following study.

### siRNA-1 with the strongest inhibitory effects was selected for ARNO blocking

**Figure 2.**
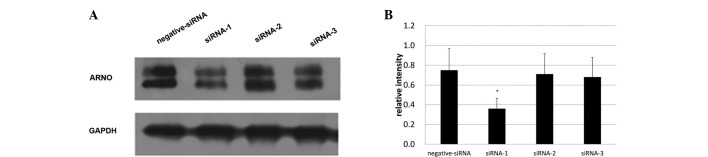
The expression of ARNO in HT-29 cells as detected following RNA interference of siRNA. (A) The expression of ARNO as detected by western blot analysis, following RNA interference in HT-29 cells. (B) Quantification of the trace density value of western blot analysis in HT-29 cells. ^*^P<0.05 for siRNA-1 vs. siRNA-2 or -3; n=3.

To select the most effective siRNA for ARNO, three siRNAs (siRNA-1, -2 and -3) were designed to inhibit the expression of ARNO. Expression of ARNO was then detected under the inhibition of these three siRNAs. The greatest inhbitory effect was produced by siRNA-1; the maximum inhibition rate was 49.271%. Therefore, siRNA-1 was selected to be the ARNO siRNA inhibitor that was used in the present study (Fig. 2). The selected ARNO siRNA sequence pair was: 5′-AGTGCATTAAAGCAGCCATCAG-3′, and 5′-TCAGTGTCGCTTCGTGGAG-3′.

### Inhibition of cytohesins reduces EGF pathway signaling in HT-29 cells

**Figure 3.**
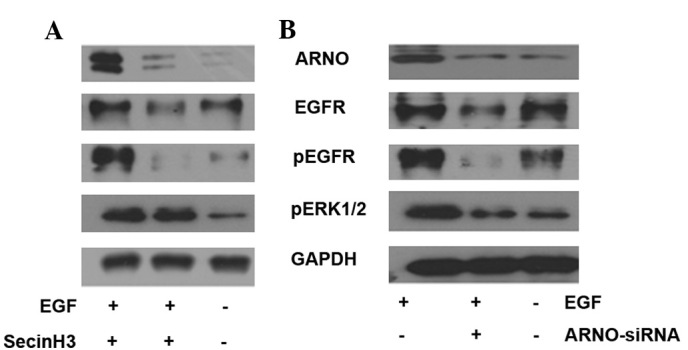
Cytohesin-2 (ARNO) enhances the activation of EGFR. (A) The cytohesin inhibitor SecinH3 reduces EGFR signaling. Western blot analysis of HT-29 cells treated with SecinH3 or solvent and stimulated with EGF, respectively, is shown. Phosphorylation of the indicated proteins was determined by immunodetection using phosphospecific antibodies. Glyceraldehyde phosphate dehydrogenase (GAPDH) served as a loading control. (B) ARNO siRNA reduces EGFR signaling. Western blot analysis of HT-29 cells treated with ARNO-siRNA or Lipofectamine 2000 and stimulated with EGF, respectively, is shown.

To detect the function of cytohesins in the EGF pathway, cytohesins were inhibited by SecinH3 and ARNO siRNA in HT-29 cells. In the assay, HT-29 cells were cultured in 35 mm glass-bottom dishes, marked as group A, B or C. All cells were cultured with 1% FBS culture medium. SecinH3 (or a mixture of 100 pmol ARNO siRNA in 5 *μ*l Lipofectamine 2000) was added to dishes from group B when cells had spread to cover 70% of the dishes for 10 h. Simultaneously, 0.2% DMSO (or 5 *μ*l Lipofectamine 2000) was added to dishes from groups A and C as a control; then 50 ng/ml EGF (Peprotech, Inc.) was added to dishes from groups A and B for 5 min. Western blot analysis was employed to test the expression of the EGF pathway-associated molecules, which included ARNO, EGFR, p-EGFR and p-ERK1/2. The results indicated that when cytohesins were blocked by SecinH3 or inhibited by ARNO siRNA, ARNO expression was reduced in HT-29 cells. Additionally, phosphorylated molecules of the EGF pathway, including p-EGFR and p-ERK1/2, were downregulated in HT-29 cells (Fig. 3).

### Blocking cytohesins inhibits the proliferation of HT-29 cells

**Figure 4.**
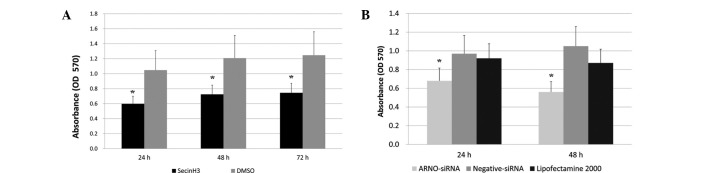
SecinH3 and ARNO siRNA inhibit the proliferation of HT-29 cells. (A) SecinH3 inhibits the proliferation of HT-29 cells. The graph shows the relative cell number (from an MTT assay) after 24, 48 and 72 h treatment with either SecinH3 or DMSO. ^*^P<0.05 for the SecinH3 blocking group vs. the control group; n=3. (B) ARNO siRNA inhibits the proliferation of HT-29 cells. The graph shows the relative cell number (from an MTT assay) following 24 and 48 h treatment with ARNO siRNA or negative siRNA. ^*^P<0.05 for ARNO siRNA vs. negative siRNA at 48 h; n=3.

To detect whether cytohesins are involved in the proliferation of HT-29 cells, we used the specific cytohesin antagonist SecinH3 and the EGFR-expressing human colorectal adenocarcinoma-derived HT-29 cells. HT-29 cells were treated with SecinH3 and then proliferation was detected by an MTT assay. DMSO was added to the cell culture medium in the control group. After culture for 24, 48 and 72 h, the inhibition rates of SecinH3 compared with the control group were 56.77, 58.72 and 57.22%, respectively (n=3, Fig. 4A).

The ARNO siRNA described previously was used as an inhibitor to identify whether ARNO downregulation is capable of reducing the proliferation of HT-29 cells. The MTT assay results demonstrated that the growth and proliferation of tumor cells were significantly inhibited by ARNO siRNA at 24 and 48 h, while the inhibition rates were 68.63 and 58.95%, respectively, compared with the Lipofectamine 2000 group (n=3, Fig. 4B).

## Discussion

Growth and survival of cancer cells is critically dependent on specific signaling molecules (14). The EGF pathway is considered to be the most prominent signaling pathway in colorectal cancer, as it regulates the differentiation, survival, proliferation and migration of cancer cells. Recently, certain individuals with wild-type Kras gene colorectal cancer have benefited from therapies targeting the EGFR. However, resistance to the EGFR blockade inevitably occurs due to a mutation in the gene encoding EGFR that impairs the binding of cetuximab to EGFR (15–17). Therefore, it is necessary to select new targets in this pathway to overcome the resistance acquired due to mutations.

Recently, Yonesaka *et al* identified acquired resistance to EGFR target therapy via increased signaling through Her2 (ErbB2; also a member of the ErbB family). Notably, the authors demonstrated that either amplification of ErbB2 or increased levels of the ErbB3/ErbB4 ligand heregulin led to *de novo* or acquired cetuximab resistance (18), and Ruan *et al* achieved similar results in a breast cancer study (19). Cytohesins, family members of GTPases, have been researched for their regulation of the reassembly of the cytoskeleton and the activation of integrin or the integrin signaling system, which is critically associated with cell adhesion and migration (11,20,21). A further study identified that cytohesins as EGFR activators may form a layer of positive regulation by facilitating the structural rearrangements required to convert the receptor dimer into its active conformation in lung cancer (10).

Studies by Kolanus (11) and Ogasawara *et al*(22) concerning the expression of cytohesins demonstrated that cytohesin-2 (ARNO) and -3 (Grp1) were ubiquitously expressed, whereas cytohesins-1 and -4 were primarily leukocyte-specific. Cytohesin-1 is a key regulator of neutrophil adhesion to endothelial cells and to components of the extracellular matrix, which may influence cell emigration through its dual opposing effect on β1 and β2 integrin activation (23). Additionally, ARNO behaves as a bistable switch, as it has an absolute requirement for activation by an Arf protein but, once triggered, it becomes highly active through the positive feedback effect of Arf1-GTP. This property of ARNO may provide an explanation for its function in signaling pathways that, once triggered, must move forward decisively (24). Additionally, in the present study, we detected the presence of cytohesins in the cytoplasm (near the membrane) by immunofluorescence, and ARNO was the most highly expressed cytohesin family member. Therefore, we employed molecular ARNO and the HT-29 cell line as subjects for the other sections of our study. Whether the strong expression of ARNO in colorectal cancer cells, potentially by enhanced EGFR signaling, contributes to tumor differentiation, survival, proliferation and migration, is yet to be determined. However, this has been identified in other types of cancer cells (25,26).

In the cell proliferation section of the present study, HT-29 cells were stimulated by human EGF in the presence of SecinH3 or treated with ARNO siRNA. As a result, both SecinH3- and ARNO siRNA-treated cells demonstrated a 56.77-68.63% inhibition rate compared with solvent-treated samples. Therefore, we hypothesize that inhibiting cytohesins contributed to the reduction in EGFR signaling. To identify the mechanism of this inhibition in HT-29 cells, i.e. whether the enhancement of EGFR activation by cytohesins was due to the effect of cytohesins on EGFR, we investigated the activation of certain EGFR pathway molecules (p-EGFR and p-ERK1/2). Our results gave support to this mechanism of inhibition.

In conclusion, ARNO, an important isoform of the cytohesin family, is highly expressed in colorectal cancer cells and enhances EGFR signaling, which contributes to tumor differentiation, survival and proliferation.
